# CFD Analysis and Optimum Design for a Centrifugal Pump Using an Effectively Artificial Intelligent Algorithm

**DOI:** 10.3390/mi13081208

**Published:** 2022-07-29

**Authors:** Chia-Nan Wang, Fu-Chiang Yang, Van Thanh Tien Nguyen, Nhut T. M. Vo

**Affiliations:** 1 Department of Industrial Engineering and Management, National Kaohsiung University of Science and Technology, Kaohsiung 80778, Taiwan; 2 Industrial University of Ho Chi Minh City, Ho Chi Minh 70000, Vietnam; 3 Thu Dau Mot University, Thu Dau Mot 75000, Vietnam

**Keywords:** volute casing design, multi-objective design, CFD analysis, computational analysis

## Abstract

In this study, we proposed a novel approach to improve centrifugal pump performance with regard to the pump head, pump efficiency, and power. Firstly, to establish constraints, an optimal numerical model that accounted for factors such as pump efficiency and the head was considered. The pump was designed, and an artificial intelligence algorithmic approach was applied to the pump before performing experiments. We considered a set of models by selecting the parameters of the centrifugal pump casing section area, the interference of the impeller, the volute tongue length, and the volute tongue angle. The weights of the factors of safety and displacement on the optimization indices were estimated. The matrix of the weights for the optimal process was less than 38% or greater than 62%. This approach guarantees a complicated multi-objective optimization problem. The results show that the centrifugal pump performances were improved.

## 1. Introduction

Centrifugal pumps are rotational devices with two essential components: an impeller and its volute casing. The impeller is the rotary component, and the volute casing is the static one. Design frameworks are imperative to instruct the designers in doing their tasks step by step. Considering the above-mentioned practical difficulties, we performed this study using a multi-optimization design approach to overcome these limitations during the design procedure. This design method is currently commonplace in not just mechanical designs but other related domains as well.

Optimum design in the mechanical field is attracting significant interest in modern research [1,2]. The specialized design requirements include smoothing out within a short time and at a low cost [3,4]. It is worth mentioning that designed products do not require the designers’ skills and experiences. The optimum design for a centrifugal pump casing usually requires two steps. Firstly, a model designed using the numerical method is used for simulation to assume the best dimensions of the structural body. In the next step, the optimal strategy is applied to obtain optimal parameters, which are the best references for practical design and manufacture. With this approach, in most cases, the optimal strategy is always considered the tool for solving global optimization problems. The improvement of centrifugal pump casing using a multi-objective is a complex problem that has been assessed in several studies so far [5,6,7,8,9]. Demeulenaere et al. [7] inspected a smoothing-out technique on centrifugal pumps using 3D models for numerical programming with many genetic algorithms. The authors tried to improve the centrifugal pump efficiency (*η*) and head (*H*) while reducing the loss of net positive suction head required (NPSHr) at the double-assorted stream rates. They also found that the edges of a centrifugal pump should have more back and forth movement compared to the central line. Many approaches for optimizing centrifugal pumps are briefly shown in Table 1.

The volute casing material and geometry of the pump were investigated by Lorett, J.A. and Gopalakrishnan, S. [20], who incorporated two major components for the most efficient mechanical execution of the centrifugal pump casing while operating in a high-pressure environment. The investigation detailed the model, the static basic assessment, and the mathematical programming alterations that were carried out to alter the volute design of the centrifugal pumps under research. The FEM (finite element method) and CFD (computational fluid dynamics) were evaluated during the initial design assessment stage without employing the optimum design. This was done to evaluate the two methods’ performance. Important mathematical updates were applied to the centrifugal pump model; nonetheless, the alterations in the mathematics programming needed to have a minor effect on the pressure-driven execution to avoid outrageously high amassing costs.

Last but not least, we performed specific investigations of volute casings using our novel method to validate enlightening results and audit water-driven performance. The multi-objective optimum procedures of the centrifugal pump with four optimum parameters (any of which could be picked by scientists or designers) were investigated and suggested by Rosu et al. [21]. Along with the parameters mentioned above, we attempted to find the best pump efficiency and head and tried to reduce pump power while performing the optimal procedure.

However, TLBO (teaching–learning-based optimization) is one of the algorithmic approaches not yet employed to designing pumps; therefore, we expected to apply this method to this study. Our approach was to define the centrifugal pump casing design to limit the analysis to its region of applicability and provide adequate pump performance. The TLBO approach was used for constraining the pump pressure distribution to one that used our assumption of the optimum centrifugal pump casing after applying the design procedure. While a better approach would be to pay the cost of sophisticated analysis to ensure the validity of the results, our optimum design took advantage of the assumptions included in the inviscid fluid dynamics to achieve mathematical improvement. Additionally, using approximation techniques improved design efficiency, as also seen early in the design of both impeller and volute casing. In practice, efficiency helped guide the early phase of the plan, but a more sophisticated analysis is essential for a reliable optimum.

In addition, the centrifugal pump volute casing is a three-dimensional model that needs to function correctly over various operating situations while maintaining its overall efficiency. At this point, the goals of the design included maximizing return on investment, requiring the least amount of energy (electricity), and meeting other composite requirements. On the other hand, it was equally apparent that a minor improvement in the design would, throughout the centrifugal pump’s lifetime, compensate for the optimization expenses several times over. In addition to that, a multi-objective optimization design has the potential to improve pump performance. The majority of research being done in this area is focused on finding a solution to this optimal design dilemma. In general, for the sake of this investigation, we did not use CFD in the design process. Instead, we relied on systematic circumstances to determine water-driven efficiency, head, and power. A cutting-edge multi-objective algorithm calculation has been utilized in several research projects [22,23,24,25] to improve pump performance and torque. The current multi-objective optimum design was constrained to reflect several trade-offs in creating the centrifugal pump. In that regard, the three-objectives optimum was considered to be any practical approach that increased the pump efficiency (*η*) and pump operational steadiness under the off-design conditions [26,27]. The water ran smoothly at the planned stream rate and the outspread push coefficients of the structure stream rate were utilized to upgrade both the pressure-driven productivity and the working steadiness of the volute casing. When using a model as the proxy model, the multi-objective optimization design is roughly estimated, as seen in [28,29,30]. The TLBO approach is an optimizer with sequential minimum and maximum techniques. It is clear that the simulation results generated the overall program flow logic for the practical design of trusses on approximation techniques. This method seems extremely powerful for all constraints presented in this study except for the safety factor limits. The performance of nonstructural masses appeared to be larger than that of structural ones. The safety factor values could confirm this once the optimizations of pressure, buckling, and reactional parameters are complete. However, precise analysis is famously relevant to structure optimization. Interestingly, several modern complicated tasks in optimization design have faced a challenging design problem.

The remaining sections of this paper are organized as follows: CFD (numerical model design) and techniques are introduced in Section 2. In this section, numerical simulation and optimal procedures that were performed are discussed. Several cases with different shapes are shown to demonstrate our explorations of a suitable casing shape model. The found parameters were assessed for the optimization process. Finally, the optimization step is illustrated in detail. Results and a brief discussion are explained in Section 3. Section 4 contains a summary and the conclusions of the research.

## 2. Model Design and Methods

### 2.1. Numerical Computation

**Figure 1:**
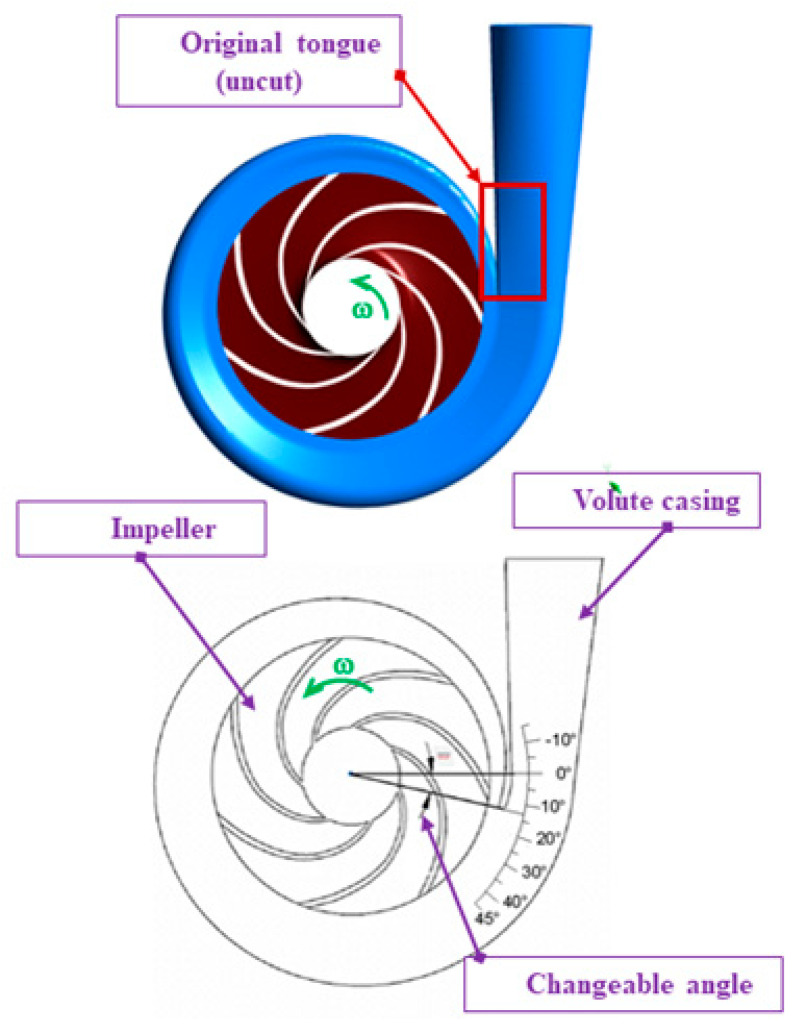
The designed volute casing of the centrifugal pump [21].

The best volute casing section area was first confirmed and then utilized to find out the best numerical volute casing for the second step in this study, after which the design with the best effectiveness point (BEP) was explored. Next, a set of CFD investigations to utilize the mass flow rate, pressure contribution, speed vectors, and so on, as well as vortex speed vectors, was employed with the diverse volute tongue to discover the reasonable tongue shape. The best volute casing in this stage was utilized for the optimum design procedure, which was our last improvement stage. Finally, the optimum designs that we found within a set of limitations are presented in Section 2. According to our data, the chosen centrifugal pump model consisted of volute casing types designed by researchers at Mokpo National University (Korea) [31]. An unstructured volute casing was generated, as shown in Figure 1. It was usable for CFD. The area section of the volute casing was formed and fixed by the four dimensions of the width (*b* = 24 mm) and the divergent angle (υ = 15°); however, the design variables (*h*) and (*R*_1_) were controlled to generate the model corresponding to the Stepnoff method of pump design. The section areas of the volute casing model at the different positions were the special plans that helped us measure the pressure boundary after simulation to collect the data. In another approach, we divided the changeable angles of the volute casing tongue that affect the tongue length dimension. Following this methodology, we tried determining the changeable angle’s effects on pump performance. We regarded this as a variable for the ideal procedure (as explained and discussed in the following sections). This study took three factors into account: the length of the tongue, the size of the volute casing, and the distance between the pump impeller and the pump volute. The following is a summary of a few centrifugal pump specifications: (i) flow coefficient: *Φ* = 0.12; (ii) head coefficient: *X* = 0.83; (iii) impeller inlet diameter ratio: *D_os_* × *D*_2_^−1^ = 0.64; (iv) impeller outlet width ratio: *b*_2_ × *D*_2_^−1^ = 0.05; (v) blade number: *z* = 6; (vi) volute diameter ratio: *D*_3_ × *D*_2_^−1^ = 1.08; and (vii) volute casing inlet width ratio: *b*_3_ × *D*_2_^−1^ = 0.09.

### 2.2. Introduction to TLBO

The following is given with the anticipation that the TLBO will be used with an intelligent progression mechanism [32].

The first step: expression of the optimal problem and instatement of the optimal factors.

Instate the resident size (*Q_n_*), generations number (*G_n_*), variables of design (*D_n_*), and cutoff points of design parameters (*Upper_L_*, *Lower_L_*).

Characterize optimal issues as Minimize *f (V)*

Subject to *V_i_* ∈ *v_i_* = 1, 2,…, *D_n_*

where *f (V)* stands for the objectives function and *X* stands for vectors of plan factors with the end goal that *Lower_L,i_* ≤ *vi* ≤ *Upper_L,i_*.

The second step: initializing the populace.

An arbitrary populace is produced as indicated by the size of the populace and plan factors. To TLBO, the size of the public demonstrates the students, and the plan factors show, for example, courses introduced. These are communicated as:

(1) $$Population=\left[\begin{array}{lll} V_{1,1} & V_{1,2} & V_{1,D}\\ V_{2,1} & V_{2,2} & V_{2,D}\\ . & . & .\\ . & . & .\\ . & . & .\\ V_{Q_{n},1} & V_{Q_{n},2} & V_{Q_{n},D} \end{array}\right]$$

The third step: the teacher phase.

Compute the mean value that offers the average for the specific course as

(2) $$Mean_{i,D}=\left[mean_{1},mean_{2},mean_{3}\ldots mean_{D}\right]$$

A great solution performs like a lecturer to the iterations:

(3) $$V_{teacher}=V_{f\left(x\right)}=min$$

The teacher attempts to lift the average through *M_i,D_* to *X_i,D_* as a teacher that acts as a neo mean for that iteration. Therefore, *Mean_new,D_* = *V_teacher,D_*. This variation among the means is described below:

*Difference_i,D_* = *r* × (*Mean_new,D_* − *TF* × *Mean_i,D_*) (4)

The results of *TF* can be chosen as 2 or 1. After calculation, it can be inserted into the present solution to update its results by utilizing

*V_new,D_* = *V_old,D_* + *Difference_i,D_* (5)

*V_new_* is accepted if it produces a better function value.

The fourth step: the learning phase.

In this step, students enhance their awareness and interactive support matures. The above section explains this computationally.

The fifth step: the termination criteria.

When the max number of generations is obtained, the process is stopped. Otherwise, repetition from the third step is carried out. In the above steps, no arrangements are made to deal with the limitations of the issue. The boundaries within the procedure include the consolidation of static punishments, dynamic punishments, versatile punishments, etc. In the proposed TLBO technique, a compelled Deb’s heuristic is utilized to deal with choice of strategy. This technique uses a competition choice administrator wherein two arrangements are chosen and contrasted. The accompanying three heuristic guidelines are actualized for their choice: (a) if only one arrangement is possible and feasible, this achievable arrangement is preferred. (b) If two arrangements are possible, the arrangement with the greater target performance is preferred. (c) If neither of the two arrangements are feasible, the arrangement with less violations of the requirements is preferred. Those principles are actualized toward the ending of the second and third steps. For example, toward the finishing stage of the teaching, during the student stage, if *V_new_* gives a better-estimated capacity than *X_new_* with regard to the completion of the second and the third step, Deb’s requirement will choose *V_new_* dependent on the heuristic standards.

### 2.3. Assessment of Efficiency Index

This part was customarily distributed throughout the study population. The algorithm evaluation rate was upgraded to verify the proposed TLBO efficiency. It was determined as the total time of running speed to the absolute results of objectives combined optimally. It was calculated by:

(6) $$R_{e}=\frac{T_{e}}{\left|f_{val}\right|}$$

where *f_val_* is the value of the combining purpose and *T_e_* is the full-time run.

### 2.4. Numerical Methodology

In this study, the calculation boundary conditions were set as follows:

(i) Water was used as the working fluid.
(ii) SST was selected as the turbulence model.
(iii) Boundary conditions were set as (a) inlet (total pressure) and (b) outlet (mass flow rate).
(iv) The interface type was set as fluid–fluid.
(v) The analysis type was set as steady-state.
(vi) The frame change and mixing model were set as frozen rotor and none, respectively.
(vii) The residual target was set at 100,000.

All statistical analyses were performed using simulation by employing the tools of ANSYS. In the selected pumps, the pump efficiency (*η*) with NPSHr was defined as a significant objective function for instantaneous optimization. This desired centrifugal pump efficiency was characterized as

(7) $$\eta =\frac{P_{out}}{P_{in}}$$

where *P_in_* stands for the charge power (or shaft power). *P_out_* is the positive power shifted via this selected centrifugal pump to the fluid. It was specified as shown in the equation below:

(8) $$P_{out}=\rho \times g\times H\times Q$$

Currently, it is a generally accepted assumption that NPSHr is a vital factor in the fluid and necessary to avoid the adversarial relationship between the draw-release nozzle and the impeller-eye, short of producing evaporation. This is an attribute of the diffusive casing and seems to be shown in the radiating casing shapes and changes with structure design, resizing, and working environments [33,34]. Increasing the NPSHr is very hazardous and may provoke a decline in performance. NPSHr remains resolved through the condition below:

(9) $$NPSHr=\frac{P_{in}-P_{out}}{\gamma }-\frac{v_{in}^{2}}{2g}$$

where *p_in_* stands for the inlet pressure, *p_min_* stands for the most negligible pressure of the entire blades, which the numerical program may define, γ and *v_in_* are the explicitly unsolidified mass and the charge velocity, respectively. To incompressible velocity, the steadiness and the momentum equilibrium equations are shown below [35]:

(10) $$\frac{\partial v_{i}}{\partial x_{i}}=0$$

(11) $$\frac{Dv_{i}}{D_{t}}=-\frac{1}{\rho }\times \frac{\partial p}{\partial x_{i}}+v\frac{\partial^{2}v_{i}}{\partial x_{i}\partial x_{{}^{{}_{i}}}}-\frac{\partial }{\partial x_{i}}\bar{u_{i}u_{j}}$$

Flow cavitation is the creation of vapor bubbles in low-pressure areas. The pressure coefficient *Cp* is often used to stand for nondimensional static pressure, *p*, in any flow:

(12) $$C_{p}\frac{\left(p-p_{1}\right)}{\frac{\rho U^{2}}{2}}$$

**Figure 2:**
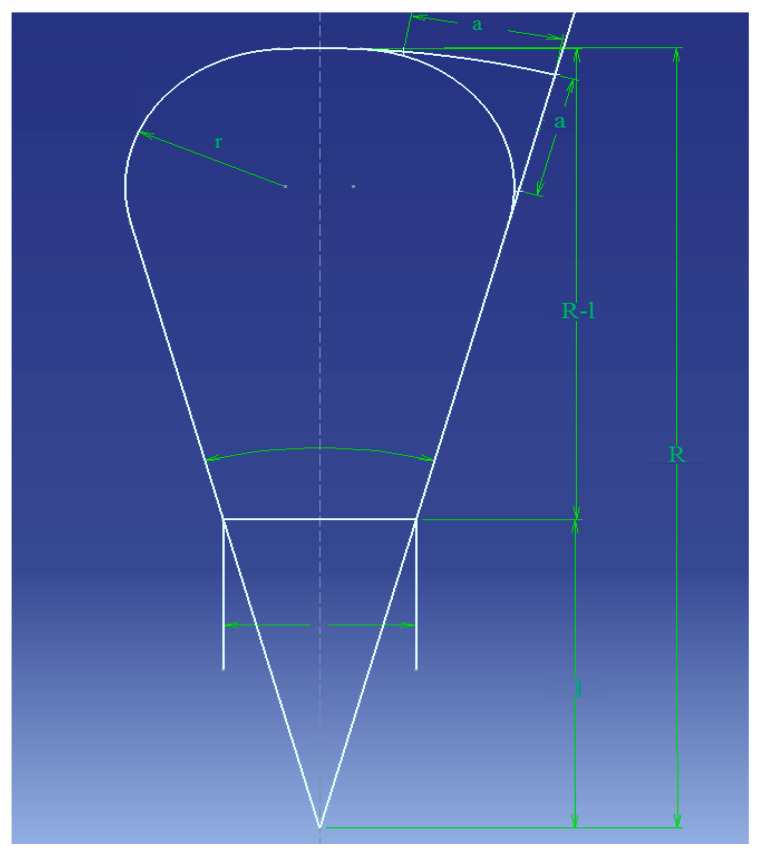
Internal cross-sectional area profile of designed volute casing.

The static pressures p_1_ and U were used as a baseline, respectively, and specific velocities were used at the charging tip speed. In the past, centrifugal pump calculations were the subject of several investigations [36,37,38]. Due to the time constraints of this investigation, we decided to exclude pump cavitation estimations. The volute casing cross-sectional area, the radius of the corners, and *R*, *a*, additional dimensions of the cross-sectional area, are shown in Figure 2.

The pump for this project was a single-stage design.

(13) $$\begin{array}{l} \frac{D_{3}}{D_{2}}=1+0.0012n_{s}^{0.87}=1+0.0012\times 127^{0.87}=1.078\\ D_{3}=1.078D_{2}=1.078\times 0.272=0.304m \end{array}$$

Select $D_{3}$ = 300 mm:

When considering the most popular and practical type of channel design round the inlet width was obtained from Equations (13) and (14):

(14) $$b_{3}=b_{2}\times 2=0.012\times 2=0.024\;m$$

Flow areas between vanes:

For this impeller and the inlet area between vanes was approximated by Equations (15)–(17):

(15) $$S_{a}=3+0.18p^{0.5}{\left(D_{s}+D_{mx}\right)}^{0.56}=3+0.18\times 3.3^{0.5}\times {\left(300+68.3\right)}^{0.56}=12\;\mathrm{mm}$$

(16) $$A_{v}=\pi D_{3}b_{3}\sin\alpha_{v}=\pi \times 0.3\times 0.024\times \sin6.5^{{}^{\circ}}=0.00256\;m^{2}$$

(17) $$v_{3}=\frac{Q}{60A_{v}}=\frac{1}{60\times 0.00256}=6.51\;m/\sec$$

where ν_3_ is the velocity of the inlet of the centrifugal pump. *S_a_*, *A_v_*, *D_s_*_,_ and *D_mx_* are other basic dimensions of the centrifugal pump.

**Figure 3:**
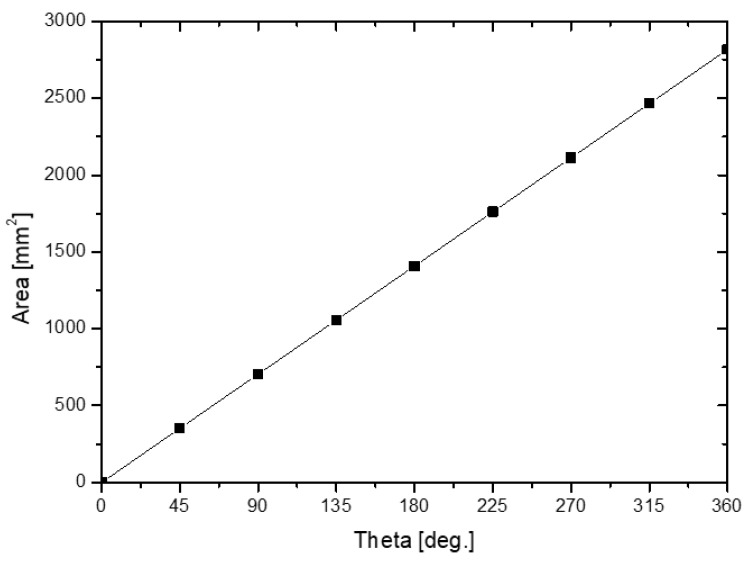
Area distribution of the volute model.

For water pumping, the right formula for the outlet area (see Figure 2, which presents the internal cross-section area profile of the designed volute casing) between vanes was computed using Equations (18)–(20). Figure 3 clearly shows the area distribution of the volute model.

(18) $$A_{s}=\frac{lR^{2}\theta }{2}-\frac{lb_{3}^{2}\cot\frac{\theta }{2}}{4}=0.262R^{2}-5.375\;({\mathrm{cm}}^{2})$$

(19) R=(As+5.3750.262)12 (cm)

**Figure 4:**
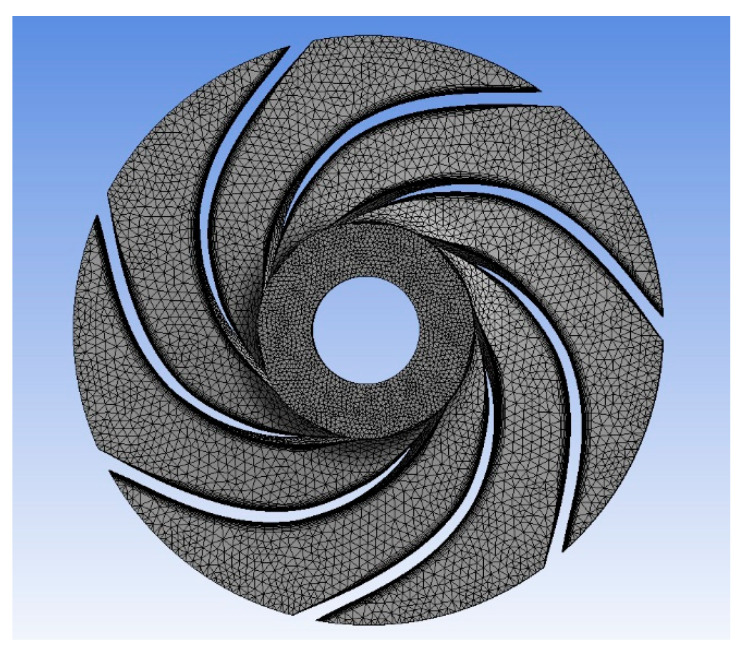
The unstructured meshes of the impeller.

**Figure 5:**
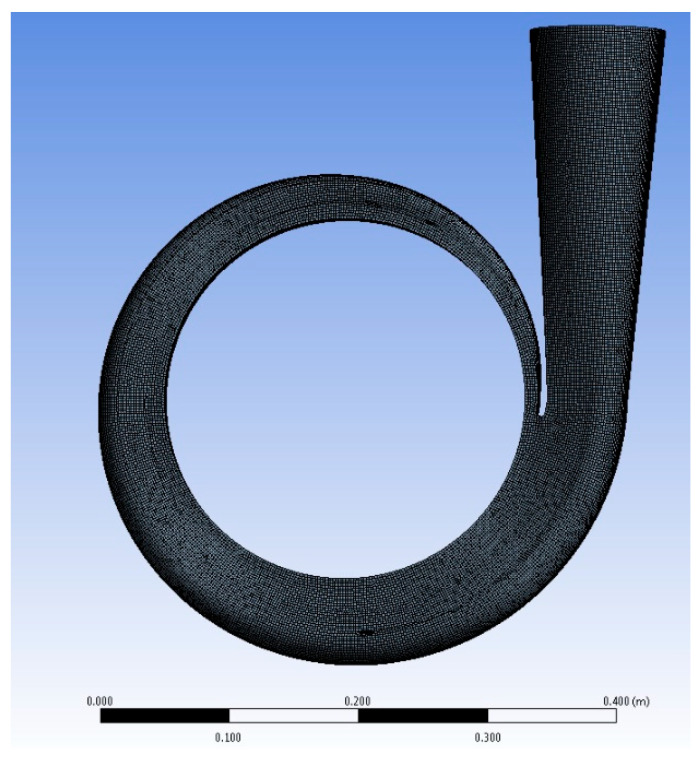
The unstructured meshes of the volute casing.

**Figure 6:**
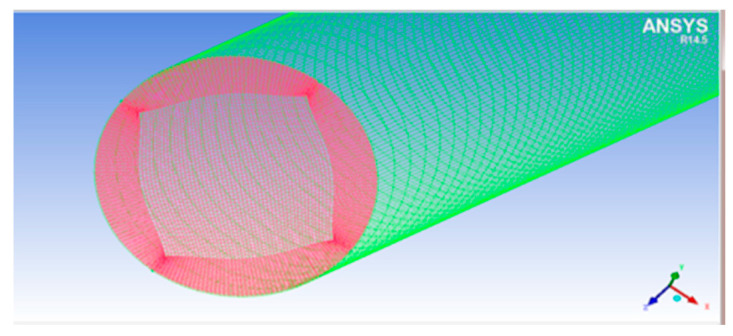
The unstructured meshes of the draft tubes (inlet and outlet pipes).

**Figure 7:**
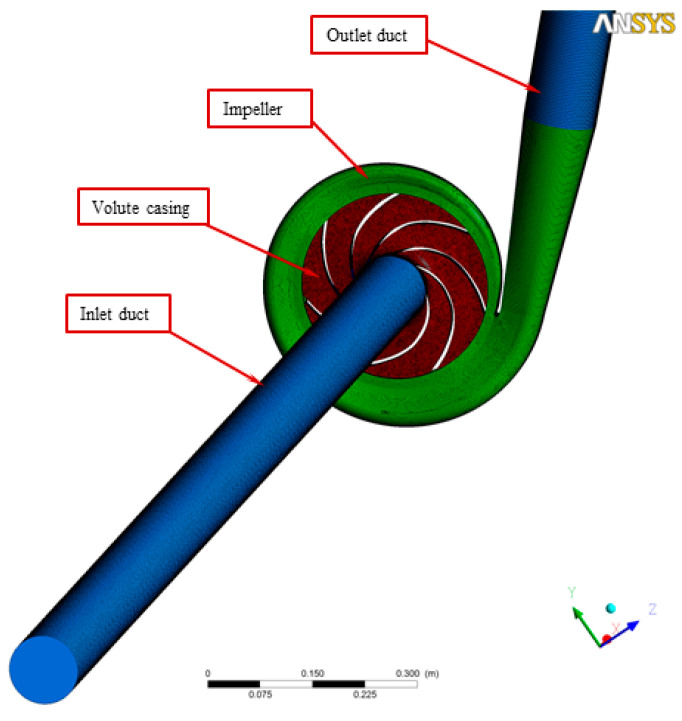
The entire unstructured centrifugal pump system for numerical simulation.

The CFD meshes of the four main parts were generated, consisting of the unstructured impeller (nodes: 0.2 × 10^6^ and elements: 2.8 × 10^6^) as presented in Figure 4, unstructured volute casing (nodes: 0.6 × 10^6^ and elements: 3.4 × 10^6^) as demonstrated in Figure 5, unstructured outlet pipe (nodes: 2.5 × 10^6^ and elements: 3.0 × 10^6^), and unstructured inlet pipe (nodes: 2.4 × 10^6^ and elements: 2.7 × 10^6^) as shown in Figure 6. The component meshes were performed using the ANSYS software. The apparatus for the simulation model was set up as shown in Figure 7. This apparatus comprised the stationary group (the outlet pipe, the inlet pipe, and the volute casing) and the rotational group (the impeller). The meshes of the impeller and the draft tubes (inlet and outlet pipes) were made from hexahedral meshes, while tetrahedral meshes were utilized for the casing. O-kind matrices close to the cutting edges and the trailing edge were utilized. The meshes were used for determining the excellent speed slope initiated through the limit layer and operated by the neighboring sturdy exteriors in the casing area. We performed mesh reliance checking for the head-coefficient for deciding the ideal hubs in every size of its calculation space. The number of hubs meshes changed in every design to examine the head-coefficient and combine with a better mesh within 0.5% of the relative error. To explore this issue, a solidified impeller interface was investigated over the course of a few distinctive comparable situations between impeller and casing, and a 12 span was chosen. These trial values standardized the computational outcomes of water-driven effectiveness and head coefficiency. It can be noticed in Table 2.

### 2.5. Process of the Optimum Design

**Figure 8:**
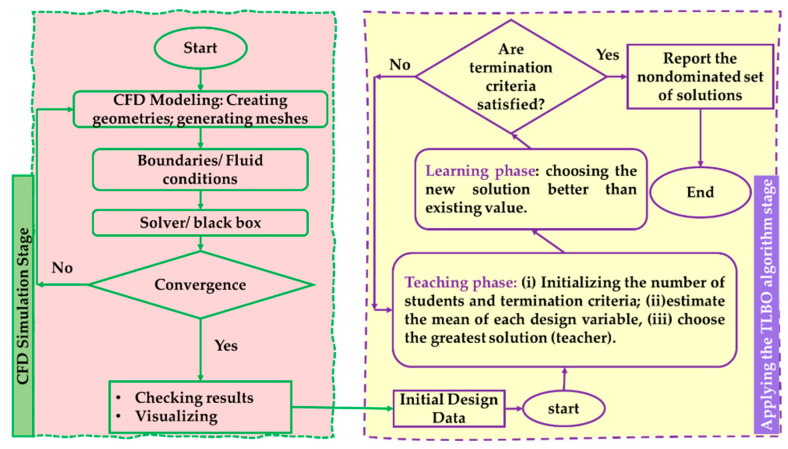
Flowchart of the optimal design.

The subject (see Equation (20) and the design variables and goal functions are established. Equation (21) provides the following description of the multi-objective optimization design process:

(i) Problem description: *min F(x)* subject to:

(20) $$LB\leq v\leq UB,\;v\in R;F(v)=\left[F_{1}(v),F_{2}(v),F_{3}(v),\ldots ,F_{m}(v)\right]$$

(ii) DOE (design of experiments): the selection of design points.

(21) $$\left\{ \begin{array}{l} Maximize\;Head\;(H)\;=f_{1}(X_{1},X_{3},\frac{X_{2}}{r_{2}},A_{o})\\ Minimize\;NPSHr\;=\;f_{2}(X_{1},X_{3},\frac{X_{2}}{r_{2}},A_{o})\\ Maximize\;efficiency(\eta )=f_{3}(X_{1},X_{3},\frac{X_{2}}{r_{2}},A_{o})\\ subject\;to:\;|\begin{array}{l} 125\leq X_{1}(\mathrm{mm})\leq 171\\ -5\leq X_{3}{(}^{o})\leq 45\\ 2\leq \frac{X_{2}}{r_{2}}(\%)\leq 12\\ 32\;\leq A_{o}({\mathrm{mm}}^{2})\leq 256\; \end{array} \end{array}\right.$$

(iii) Numerical analysis using the CFD and CFX: calculation of objective functions at each experimental point.
(iv) Surrogate modeling: it is clear to see that TLBO is constructed for objective functions.
(v) Multi-objective genetic algorithm: TLBO [36,37,38]; and
(vi) Pareto-optimal forward-facing: illustration of resolutions in function space. Therefore, to examine the ideal execution of the centrifugal pump, the pump models are used in the three-objective streamlining optimum [39,40,41,42]. The three-objective optimum problem is well-defined and explained in the following Equation (21). Figure 8 presents the flowchart of the optimal design procedure for a centrifugal pump.

The couple clashing objectives in the investigation are efficiency (*η*) and NPSHr, which are determined and simultaneously upgraded for the planned variables of tongue length ($X_{1}$), changeable angle ($X_{3}$), impeller radius divided by the gap (the distance from the impeller to the pump tongue of the pump volute) $\left(\frac{X_{2}}{r_{2}}\right)$ (determination of input parameters [31]), and the area of cross-section of the volute casing ($A_{o}$) at 0°.

## 3. Results and Discussion

**Figure 9:**
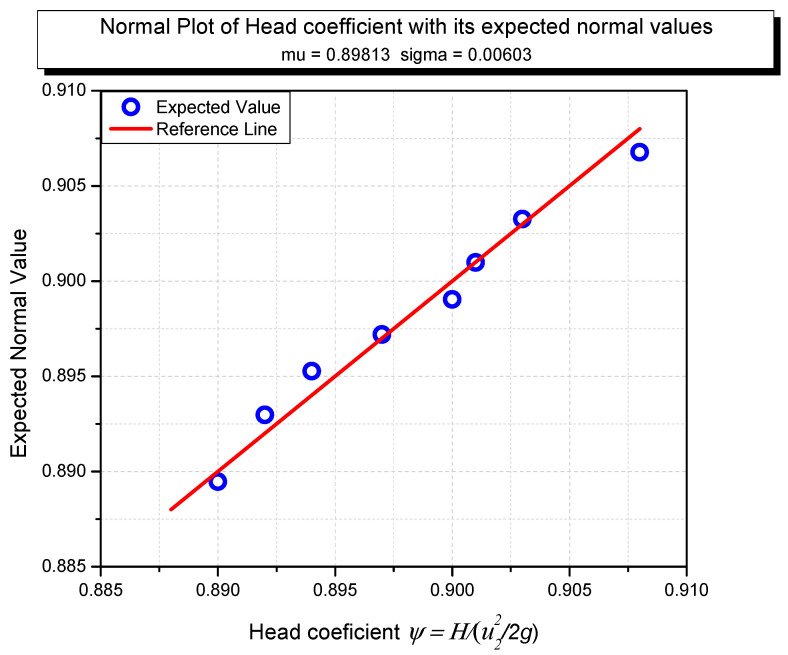
Head coefficient and its expected average values.

The absolute charge pressure was assessed upstream of the impeller channel. The source pipe stream rate was evaluated, and the downstream of the volute source was processed using ANSYS software [43,44,45,46,47,48], in which the best path from the charging gate to the discharging gate is the one with the smoothest fluid flow. Limiting the value of NPSHs, the objective work related to water-driven execution was negative on water-controlled capability. The part affected by static mass at the impeller outlet was assessed as the winding force governed via coordinating the circumferential weight dispersion. The Y+ wall in this study was less than 1. According to experts, this value is suitable for unstructured models such as ours. The head coefficients and their predicted typical values are shown in Figure 9.

**Figure 10:**
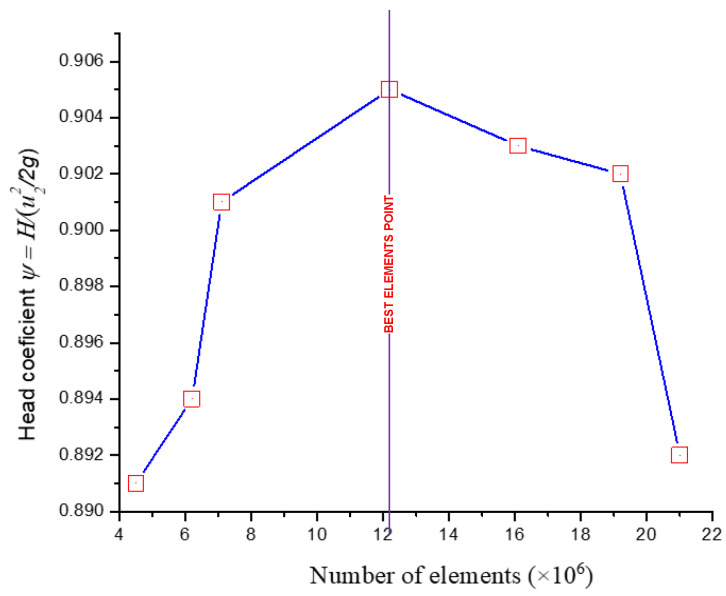
Testing independent mesh in this design.

Near the reference line, the predicted values are clustered together. The data collected in this study are expected to introduce a guideline and references for further research on pump performance. This step should be conducted before testing the independent mesh. Figure 10 shows the results of convergence and meshing independence research. The cross-sectional freedom appears critical and essential to get a handle on. This step can be applied as a form of contrast on the off chance that the arrangements the scientist expects to have are free of the meshing goal. It additionally permits the researchers to guarantee they are accomplishing the ideal arrangement time and not growing their cross-sectional and arrangement times. The best strategy to execute a mesh independence check is to take it little by little, refine and coarsen the cross-section in the significant locales, and afterward, look at the eventual outcomes and plot their distinctions. Preferably, we hope to accomplish a value that does not change excessively without over-refining the cross-section. As reference, finding the harmony between network thickness and the final arrangement is urgently vital to a CFD reenactment. A meshing independence study is an initial step to assessing the precision of our outcomes. The result proposes that the full meshes should be applied in unstructured models at 12.2 × 10^6^ elements. Therefore, the models were designed as follows in this proposal.

**Figure 11:**
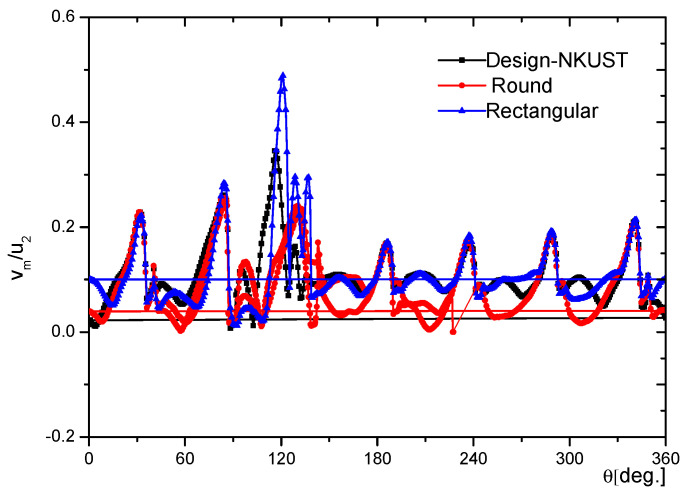
Static pressure coefficient at the volute inlet to three cross-sectional shapes.

**Figure 12:**
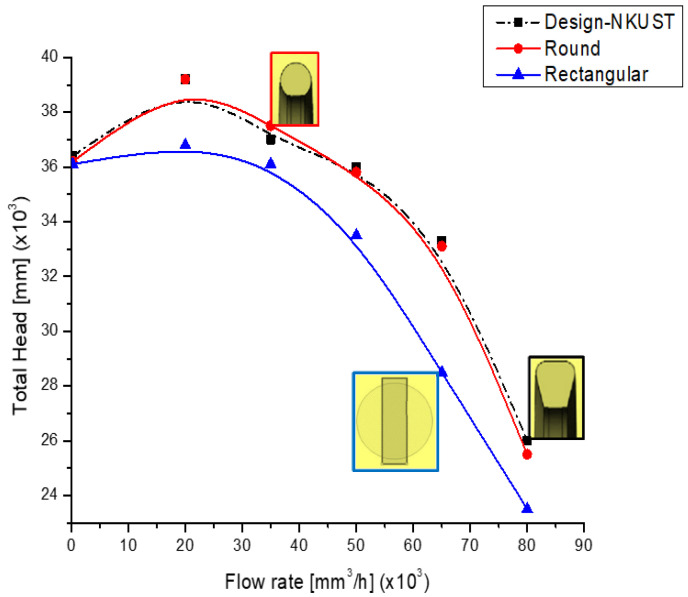
The comparison of three types of volute casing (cross-section).

Following the pump theory, we saw that when the volute casing static pressure increased, the pump performance improved. However, the very high pressure at any specific position could cause cavitation in the pump, a problem that became more serious with significant temperature increases. Figure 11 shows the static pressure coefficient at the intake of the volute for three different cross-sectional configurations. Three styles of volute casing are compared in Figure 12 (cross-section). This research determines which volute form is the ideal one to construct. Firstly, we chose the best model for our project. Figure 12 clearly shows that the geometry of the volute casing significantly impacts the efficiency of the centrifugal pump.

Furthermore, we saw that the shape that gave the lowest pumping efficiency was the rectangular cross-sectional shape. The casing shape that offered the highest efficiency was the volute casing, whose cross-section was a rounded trapezoid at the large end (Design-NKUST). The volute casing with a circular cross-section had higher efficiency than the rectangular section but was more visible than the volute casing with a trapezoidal cross-section. Therefore, in this study, from the results shown in Figure 11 and Figure 12, we designed an unstructured centrifugal pump model according to the volute casing (Design-NKUST).

From these outcomes, the confirmed arrangements of CFD investigations [49,50,51,52,53,54,55] with various cases were directed to set up the remarkable models before applying the optimum procedure. In the previous studies, it is clear that the best correlations with the two sorts of the volute casing were re-designed with the round shape of the volute casing section. The other designs with the trapezoid shape were not chosen in this investigation. In [31], we presented the predicted performance of the centrifugal pump, which was the initial design. The line chart shows that pump performance was best at the flow coefficient of 0.026. Therefore, this best efficiency point (BEP) was the best reference for the first pump design step. After this investigation, the pump moved to another continuous step to check the effect of changeable angles on the pump performance.

**Figure 13:**
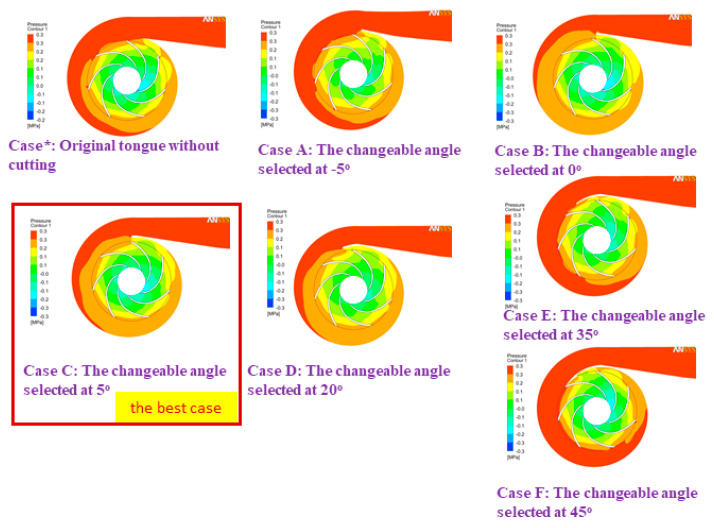
A comparison of the pressure spectrum of different angles.

**Figure 14:**
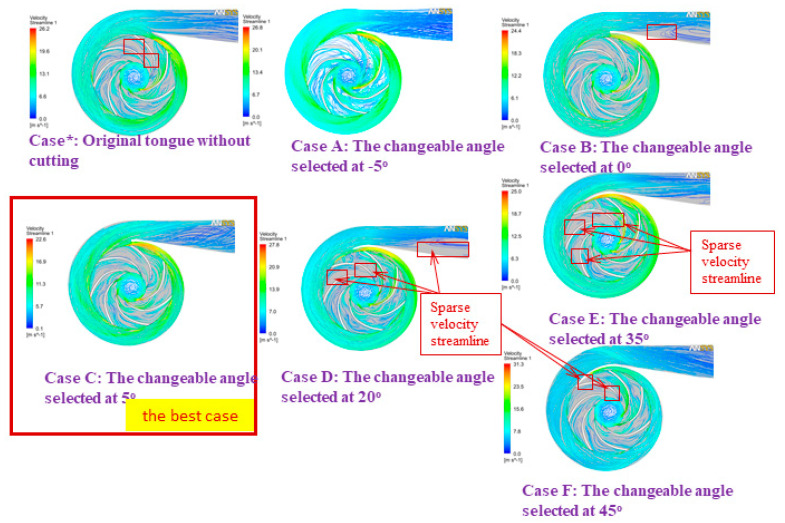
A comparison of the velocity streamlines at different angles.

**Figure 15:**
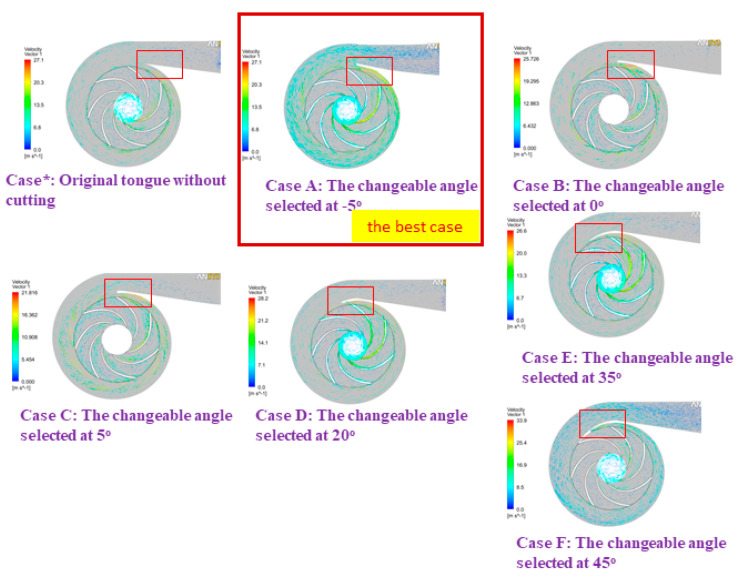
Velocity vectors of various angles in comparison.

According to [31], we demonstrated that the most effective operation of the pump took place at an adjustable angle of 5 degrees; hence, the findings of this inquiry were used in developing the numerical model for the optimization design stage. Figure 13, Figure 14 and Figure 15 display comparisons of the pressure spectrum, velocity streamlines, and velocity vectors for several selected different angles: Case*: initial design, uncut; Case A: the changeable angle of −5 degrees; Case B: the changeable angle of 0 degrees; Case C: the changeable angle of 5 degrees; Case D: the changeable angle of 20 degrees; Case E: the changeable angle of 35 degrees; and Case F: the changeable angle of 45 degrees.

Case C is used in Figure 13 and Figure 14 to demonstrate the best performance of the pump. On the other hand, it seems from Figure 15 that the centrifugal pump would operate most effectively with Case A. However, after much deliberation, it was determined that the design process of Case C produced the centrifugal pump with the highest efficiency level. To test how effective the suggested TLBO computation was, numerical simulation results for each of the three distinct goal functions were used. For all viable test frameworks, the population size (NP) and the most extreme emphasis (*N_max_*) were 50 and 100, respectively. In each of these cases, the TLBO was calculated multiple times using various arbitrarily created starting arrangements, and the best results were recorded for assessment.

The proposed TLBO method was used in the initial situational research to reduce the power loss of the centrifugal pump. The volute casing pressure allowed for the identification of this loss. In subsequent analysis [27,28,31], including ideas for different technologies, these were introduced as a whole, taking into account this critical negative impression of this proposed technology and other optimum design methodologies. With the TLBO technique, quality performance indicative of excellent design was noted.

Because other technology replication fields are unavailable and different optimization methodologies have not been documented in this work, it is impossible to compare the computational productivity of CPU cycles in the TLBO algorithm. However, the reformulation findings demonstrate the computational effectiveness of the TLBO methodology.

**Figure 16:**
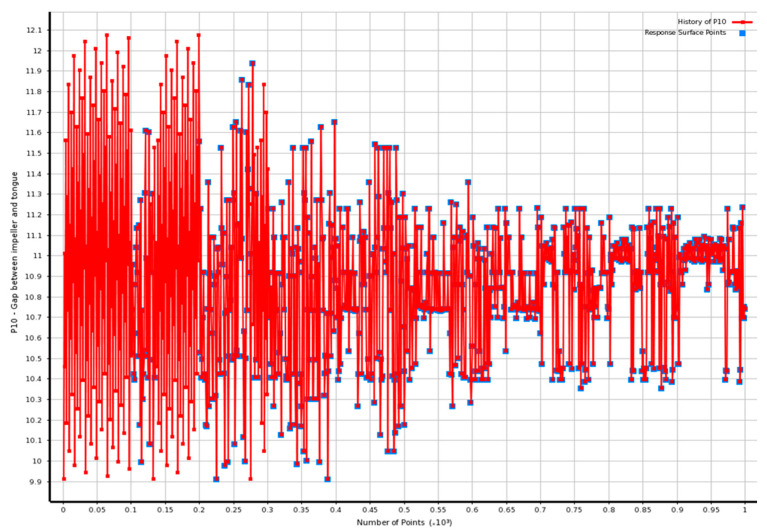
The number of points of tongue length.

The number of parameters (such as tongue length, the distance between the volute casing and the impeller, and the area of the volute section) configured in the program can be seen before running/searching for the ideal values. The snapshot of the optimization tools is shown in Figure 16. Using the optimal method, we can see that it demonstrates how the computational program, and its consequences are intertwined. The length of the tongue points is correlated with the following variables. The next choices were made for the parameter and its levels:

The section-area of the volute casing at 0°, *A_o_* (cm^2^) shows a level 1 of 0.32, level 2 of 1.44, and level 3 of 2.56 cm^2^;

The tongue length, *X_2_* (mm), including level 1 with 125.3, level 2 with 148.2, and level 3 with 171.1;

The ratio between the gap and the impeller radius. $\frac{X_{2}}{r_{2}}$ (percent) has a level 1 of 2.1, level 2 of 7.1, and level 3 of 12.1;

Changeable angle, $X_{3}$ (degree) including level 1 of −5; level 2 with 20, and level 3 of 45. In can be noticed in Figure 16.

Exploring this investigation, we found that the centrifugal pump performance was factually determined. Our data show that the more bent the volute tongue shape is designed, the higher the increments in centrifugal pump efficiency. This problem happens because as the volute tongue bends increasingly, it appears to form a point at its pinnacle. Subsequently, it is average in quality but easily worn, making it difficult to manufacture and maintain. The improvement of pump efficiency itself improves, particularly at dense streamlines. When designing the volute casing, the changeable tongue angles should be considered. Therefore, tongue C (Case C) achieves the best efficiency and tongue F (Case F) is the least efficient, as shown in Figure 13, Figure 14 and Figure 15.

**Figure 17:**
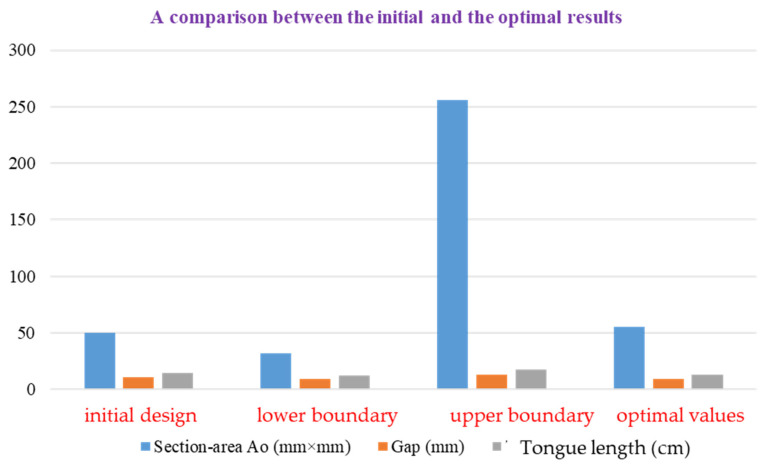
A results comparison of the initial and optimal design.

The analyses of the information introduced in this study elicit the proposed optimal methodology and show definite outcomes after optimization design. Figure 17 indicates that the highest efficiency was attained at 55 mm^2^ with a tongue length of 126 mm, a distance between the impeller and the volute casing of 9 mm, and the best volute segment territory at 0 degrees.

## 4. Conclusions

During this research project, we came up with a revolutionary method, which was then put into action and used for the volute casing of centrifugal pumps. The operation of the centrifugal pump was made more efficient. A case study with excessive pressure was selected to evaluate how useful the proposed model was. In addition to that, the objective functions were improved. The post-processing of the CFD data revealed that the applied geometrical adjustments led to a more uniform flow across the flow passageways of the best-fitting centrifugal pump components. This was demonstrated by the fact that the flow was more efficient. This optimization method can be a helpful guide for other pumps aiming to improve their performance or perhaps lower the number of pressure fluctuations they experience. During operation, this ideal design also contributed to a reduction in the amount of energy or power that was lost. The result illustrates that it is possible to implement the given technique to solve the design optimization problem. The association between this method’s practicality and application is more robust than the other alternatives.

Additionally, the effects of the different cross-sectional volute shapes on the curves were as follows: (a) the static pressure head distribution within the volute casing was almost the same for each flow rate regardless of the model, but had a constant tendency at the design flow rate; (b) the asymmetry of the spiral casing affected impeller inlet flow regardless of the volute casing types; (c) the dynamic pressure head distribution had almost the same shape, regardless of the model, depending on the flow rate from the casing and the neck to the outlet, and the trend was opposite to the static pressure head distribution within the casing and decreased from the neck to the outlet, being collected at one point.

However, the TLBO technique was only employed by one educator to enhance the results of a single class involving many students. In addition, the training factor utilized to calculate TLBO was 1 or 2. This result means the student must adjust for the instructor percentage or zero. This problem slows down the convergence speed of large-scale nonlinear optimization problems. Therefore, some changes can be considered to speed up the search process and increase the convergence speed to help reduce the costs and cut down on the time of the computation process. The findings of this search can be referenced for an optimized and improved design for bidirectional electrohydrodynamic pumps [55] and axial piston pumps [56] or other pumps.

For future works, we will fabricate the last version of this optimized centrifugal pump and experiment to verify the simulation results.

## Figures and Tables

**Table 1:** A review of optimal design methods for centrifugal pumps.

[Ref.]	Author/(s)	Design Proposal [Traditional Methods]	Design Approach [Modern Methods]	Pump Component/(s) or Others	Results
[10]	Wu, T.X., et al. (2022)	x	ANN-GA	The diffuser of the multistage centrifugal pump	Head: +1.47 m Loss: –1.89
[11]	Siddique, M.H., et al. (2022)	Computational fluid dynamics -CFD/in-house surrogate-based optimization code/experiment	x	Impeller blades	Head: +8.2% Overall efficiency: +3%
[12]	Shi, Y.F., et al. (2022)	CFD/experiment	Taguchi algorithm	Structural parameters	NPSH: 0.95% Efficiency: 61.5%
[13]	Peng, C.C., et al. (2022)	Numerical simulation and experiment (DOE, RSM)	Multi-objective genetic algorithm	Impeller and diffuser	Pressure increment: +38 kPa Efficiency: improved significantly
[14]	Parikh, T., et al. (2022)	3D RANS 1-phase turbulent simulations, CFD	Non-dominated sorting genetic algorithm (NSGA-II)	Inducer geometrical parameters	Blade: maintained short (in terms of length, sweep angle, tip clearance gap, and thickness). High hub taper angles, three blades proposed for improved pump performance
[15]	Fracassi, A., et al. (2022).	CFD	Genetic algorithm	Shape optimization	Rotational speed affecting the flow rate; uncertain results
[16]	Abdolahnejad, E., et al. (2022)	Numerical simulation tools, experiment	Methods of surface response, genetic algorithm	Impeller blades	Pump head: +2 m Efficiency: upkeep
[17]	Zhang, R. H., et al. (2021)		Knowledge mining method, NSGA-II and RBF hybrid algorithm	Pump blade shape	Hydraulic loss: smaller Efficiency: higher
[18]	Xie, X., et al. (2021)	(CFD); (FSI) fluid-structure interaction	GMDH, NSGA-III & TOPSIS	Impeller	Maximum equivalent stress of blades: reduced Efficiency: improved
[19]	Onder, A., et al. (2021)	CFD	Bees algorithm	Impeller sidewall gaps	Computational costs: reduced Efficiency: +20%
	Our proposed method	CFD	TLBO algorithm	Area of cross-section volute casing, impeller side wall gap, volute casing tongue	Improved pump performance, computational cost, and decreased time

**Table 2:** Internal cross-sectional area profile of volute casing.

$\theta^{0}$	$A_{s}$ $\left(cm^{2}\right)$	$A_{s}+5.375$	$\frac{A_{s}+5.375}{0.262}$	R (cm)	R − l (cm)	$a^{2}$ $\left(cm^{2}\right)$	a (cm)
360	28.16	33.535	127.99	11.31	6.83	2.56	1.600
315	24.64	30.015	114.56	10.70	6.22	2.24	1.495
270	21.12	26.495	101.12	10.05	5.57	1.92	1.385
225	17.60	22.975	87.69	9.36	4.88	1.60	1.265
180	14.08	19.455	74.26	8.62	4.14	1.28	1.130
135	10.56	15.935	60.82	7.80	3.32	0.96	0.980
90	7.04	12.415	47.39	6.88	2.40	0.64	0.800
45	3.52	8.895	30.95	5.83	1.35	0.32	0.565
